# Adjunctive sub-tenon triamcinolone with Susanna glaucoma drainage device: a randomized clinical trial

**DOI:** 10.1016/j.clinsp.2026.101221

**Published:** 2026-10-30

**Authors:** Marcus Vinicius Takatsu, Karin Chiaradia Takatsu, Kaio Santos Soares, Kayo Pinheiro Esposito, Leonardo Eleuterio Ariello, Renato Antunes Schiave Germano, Marcio Henrique Mendes, Aline Domingos Pinto Ruppert, Marcelo Hatanaka

**Affiliations:** a Department of Ophthalmology, Hospital das Clínicas, Faculdade de Medicina da Universidade de São Paulo (HCFMUSP), São Paulo, SP, Brazil; b Fundação Altino Ventura (FAV), Recife, Pernambuco, Brazil

**Keywords:** Glaucoma, Glaucoma drainage implants, Intraocular pressure, Triamcinolone acetonide, Ocular hypotony

## Abstract

**Purpose:**

To evaluate the efficacy and safety of Adjunct Triamcinolone (TA) with the Susanna drainage device for refractory primary open-angle glaucoma, to our knowledge, the first such evaluation with a non-valved glaucoma drainage device.

**Methods:**

This prospective, multicenter, randomized, double-blind (patient and outcome assessor) trial included 39 eyes with refractory primary open-angle glaucoma, divided into a control (n = 20) and a triamcinolone group (n = 19). Primary outcome was mean Intraocular Pressure (IOP) at six months. Secondary outcomes included success rates and postoperative complications.

**Results:**

Baseline characteristics and mean preoperative IOP were similar (24.45±8.03 mmHg control vs. 24.95±6.80 mmHg TA, p = 0.83). At six months, both groups showed similar mean IOP reduction (14.05±3.99 mmHg control vs. 14.33±4.70 mmHg TA, p > 0.99), with percentage reductions of 42.53% and 42.56%, respectively. Hypotensive eyedrop use also decreased similarly (1.57 control vs. 1.16 TA, p = 0.95). Qualified success (IOP 5‒21 mmHg) was 90% (control) and 89.47% (TA) (p > 0.05). However, the triamcinolone group had a significantly higher incidence of late-onset hypotony (52.6%vs. 0%, p < 0.001). Clinically significant hypotony requiring surgical re-intervention occurred in 36.8% of the TA group versus 0% in the control group (p = 0.003).

**Conclusion:**

In patients undergoing Susanna GDD implantation, adjunctive sub-Tenon triamcinolone provided no additional IOP-lowering benefit but was associated with a significantly increased risk of serious hypotony-related complications. Our findings do not support the use of sub-Tenon triamcinolone (20 mg) as an adjunct to non-valved GDDs in this setting.

## Highlights

• First randomized trial of subtenon triamcinolone with a non-valved glaucoma device.
• Adjunctive triamcinolone did not improve IOP control at 6 months.
• Significantly higher hypotony-related complications observed with triamcinolone.
• Trial terminated early by DSMB recommendation for safety reasons.

## Introduction

Glaucoma is the leading cause of irreversible blindness and the second leading cause of blindness worldwide.^1^ It is estimated that by 2040, 111.8 million people globally will have glaucoma.^2^ The cornerstone of glaucoma management is the reduction of Intraocular Pressure (IOP) to prevent the onset and progression of Open-Angle Glaucoma (OAG).^3^

Initial glaucoma management typically involves medical therapy with eye drops. Recently, evidence from the LIGHT Trial supports the use of Selective Laser Trabeculoplasty (SLT) as an effective first-line therapy.^4^

Despite these initial treatments, when IOP control is inadequate or patient adherence is poor, surgical intervention becomes necessary.^5^ For advanced glaucoma patients, the primary surgical options are Trabeculectomy (TREB) and Glaucoma Drainage Device (GDD), with trabeculectomy generally being the first-line treatment, and GDDs reserved for cases in which trabeculectomy has failed.^5^

The use of GDDs has been increasing, driven by recent studies demonstrating IOP control comparable to TREB. GDDs are particularly indicated for refractory glaucoma or in situations where trabeculectomy has a higher likelihood of failure, such as in neovascular and uveitic glaucoma.^5^ In Brazil, the cost is a significant barrier to the widespread use of GDDs. Within this context, the Susanna drainage device emerges as a more cost-effective alternative, providing similar outcomes to other available devices such as Ahmed and Baerveldt.^6^^,^^7^

Despite their widespread adoption, the hypertensive phase following GDD implantation poses a significant challenge, especially for patients with advanced OAG, as postoperative IOP spikes are highly undesirable. Recent studies have indicated that the adjuvant use of intraoperative sub-Tenon Triamcinolone Acetonide (TA) can mitigate the hypertensive phase, reduce peak IOP levels, and lower mean IOP for up to one year in cases involving valved GDDs implantation (Ahmed).^8^^,^^9^

While these findings with valved devices are encouraging, a critical gap exists in the literature regarding the impact of TA on non-valved GDDs, which possess distinct hydrodynamic properties and mechanisms of capsule formation. To our knowledge, no prior studies have specifically evaluated the use of triamcinolone as an adjunct to non-valved glaucoma drainage devices. Therefore, this study pioneers an investigation into the efficacy and safety of intraoperative sub-Tenon’s triamcinolone adjunctive to glaucoma surgery using the Susanna drainage implant, a non-valved device.

## Methods

### Setting and design

This prospective, randomized, double-blind (patient and outcome assessor), interventional study was conducted at the Hospital das Clínicas, Faculdade de Medicina da Universidade de São Paulo (HCFMUSP), São Paulo, Brazil, and Fundação Altino Ventura (FAV), Pernambuco, Brazil, from January 2021 to June 2022. This trial was registered at the Brazilian Registry of Clinical Trials (ReBEC), identifier RBR-3mcfsb9.

### Inclusion and exclusion criteria

Patients aged over 18-years with refractory primary open-angle glaucoma scheduled for implantation of the Susanna drainage device were assessed for eligibility. Refractory glaucoma was defined as inadequate IOP control despite maximum tolerated medical therapy and previous trabeculectomy failure. Exclusion criteria included concurrent procedures such as pars plana vitrectomy or cataract surgery, previous drainage implant and glaucoma secondary to other causes, such as neovascular glaucoma, uveitic glaucoma or glaucoma following corneal transplantation.

### Informed consent and ethical considerations

The study adhered to the principles of the Declaration of Helsinki and was approved by the Research Ethics Committee of Faculdade de Medicina da Universidade de São Paulo (CAAE 39202020.4.0000.0068; approval n° 4.488.101) and by the Research Ethics Committee of Fundação Altino Ventura (CAAE 39202020.4.3001.5532; approval n° 5.284.380). Written informed consent was obtained from all study participants prior to enrollment, following an explanation of the procedure and the nature of the study.

### Preoperative evaluations

Preoperatively, all subjects underwent a comprehensive ophthalmic examination; including Best-Corrected Visual Acuity (BCVA), slit-lamp biomicroscopy, Goldmann applanation tonometry, gonioscopy, and fundus examination. The number of glaucoma medications was documented.

### Randomization and masking

Patients were randomly assigned in a 1:1 ratio to either the control or triamcinolone group. The randomization sequence was generated using a computer-based random number generator (random.org) by an independent researcher not involved in patient recruitment or assessment. Allocation concealment was achieved using sequentially numbered, opaque, sealed envelopes containing the treatment assignment (revealed through a QR code within the envelope). The envelope was opened intraoperatively by a non-masked surgical assistant only after the glaucoma drainage device was fully secured and immediately before conjunctival closure, to determine group allocation for the triamcinolone or sham injection. Patients and outcome assessors (responsible for all postoperative data collection) remained blinded to the treatment allocation throughout the study duration. The allocation code was broken for data analysis only after the database was locked.

### Intervention

All procedures were performed by a single experienced glaucoma specialist at each center under peribulbar or retrobulbar local anesthesia, using a standardized surgical technique. The surgical field was prepared and draped in the usual sterile fashion. After inserting an eyelid speculum and irrigating with 5% povidone-iodine solution, a 7–0 silk traction suture was placed to rotate the globe and achieve optimal superotemporal exposure. A limbus-based conjunctival peritomy was created approximately 4-mm posterior to the limbus, and the Tenon capsule was dissected. The Susanna drainage implant plate was primed by gently irrigating the tube with balanced salt solution. The tube was ligated with a 7–0 polyglactin suture and two slit-like fenestrations were created anterior to the ligature. The plate was fixed to the sclera 6 mm posterior to the limbus with 2 interrupted 7–0 silk sutures. A rectangular partial-thickness scleral flap (approximately 4 × 4 mm) was created. The tube was cut to the appropriate length with the bevel facing forward and inserted into the anterior chamber through a corneoscleral track created with a 25 G needle under the scleral flap. The tube was secured to the episclera with a 10–0 nylon mattress suture. The scleral flap was sutured with releasable or permanent 10–0 nylon sutures, covering the tube. In the TA group, 20 mg (0.50 mL) of triamcinolone acetonide (40 mg/mL) were injected into the sub-Tenon space around the implant plate using a blunt hydrodissection cannula. In the control group, a sham injection procedure was performed without fluid administration. The conjunctiva and Tenon capsule were then closed with 7–0 polyglactin sutures.

### Follow-up

All patients were examined on the first postoperative day. The postoperative regimen included topical ciprofloxacin 3.5 mg/mL eye drops 4-times a day for 1-week and topical dexamethasone 1 mg/mL eye drops. Dexamethasone was administered six times daily for the first four weeks, followed by a gradual tapering regimen over the subsequent four weeks: reducing to four times daily for one week, then three times daily for one week, then twice daily for one week, and finally once daily for one week, totaling eight weeks of topical corticosteroid therapy. Follow-up examinations were conducted weekly during the first month and subsequently at 2-, 3-, and 6-months postoperatively. In addition to protocol visits, patients were assessed at approximately 6-weeks postoperatively to monitor tube opening following 7–0 polyglactin suture dissolution. IOP was measured using a calibrated Goldmann applanation tonometer at approximately the same time of day for each visit. A masked examiner obtained all measurements. The initiation of additional hypotensive eye drops was at the discretion of the treating (unmasked) glaucoma specialist targets, visual field status, and optic disc appearance.

### Outcome measures

The primary outcome measure was mean IOP at the 6-month follow-up visit. Secondary outcome measures included the proportion of eyes achieving specific IOP reduction targets, change in mean IOP from baseline, BCVA (converted to logMAR), occurrence of the Hypertensive Phase (HP), number of additional glaucoma medications, surgical success rates, and the incidence and nature of postoperative complications. HP was defined as an IOP greater than 21 mmHg on at least two consecutive follow-up visits after the first postoperative week, or any IOP elevation requiring intervention (e.g., additional medication, needling, or surgical revision), not resulting from implant malfunction or tube obstruction/retraction. Hypotony was defined as IOP < 6 mmHg. It was classified as early (≤ 30-days) or late (> 30-days). Clinically significant hypotony was defined as hypotony associated with complications (e.g., shallow anterior chamber, choroidal effusion, hypotonic maculopathy) or any hypotony requiring surgical intervention. Serious complications were defined as sight-threatening events (e.g., malignant glaucoma) or those requiring return to the operating room.

Other complications such as endophthalmitis, wound dehiscence, hyphema, implant exposure, tube erosion, encapsulation, and significant visual loss (≥ 2 Snellen lines) were prospectively recorded. Success at 6-months was defined by two criteria: Criterion I (IOP ≥ 6 and ≤ 21 mmHg) and Criterion II (IOP ≥ 6 and ≤ 18 mmHg). Success was subclassified as complete (without medication) or qualified (with medication).

### Sample size and statistical methods

The sample size was initially calculated to detect a 3 mmHg difference in mean IOP at 6 months between the study groups, assuming a common standard deviation of 3.5 mmHg, with a two-sided alpha of 0.05 and 80% power. This yielded a target sample size of 24 eyes per group (total 48 eyes). However, due to a significantly higher rate of serious complications observed in the triamcinolone group, enrollment was prematurely terminated by recommendation of the Data Safety and Monitoring Board (DSMB) after 39 eyes (20 in the control group, 19 in the triamcinolone group) had been enrolled and completed the 6-month follow-up. Statistical analyses were performed on this final sample. Descriptive statistics are presented as mean ± Standard Deviation (SD) for continuous variables and as counts (percentages) for categorical variables. Between-group comparisons for continuous data were made using Student’s *t*-test or Mann-Whitney *U test*, as appropriate based on data distribution. Categorical variables were compared using Chi-Square test or Fisher’s exact test. Repeated IOP measurements were analyzed using a mixed-effects model or Two-Way ANOVA for repeated measures. A p-value <0.05 was considered statistically significant. Time-to-event analysis for the occurrence of hypotony was performed using Kaplan-Meier survival curves, and the survival distributions were compared between groups using the Log-Rank test.

## Results

The flow of participants through the trial is detailed in Fig. 1. Between January 2021 and June 2022, a total of 90 patients were assessed for eligibility. Of these, 48 were randomized to a treatment group. Enrollment was terminated prematurely for safety reasons after 39 of the 48 randomized patients had received the allocated intervention and completed the six-month follow-up. The final analysis included 20 patients in the control group and 19 in the triamcinolone group. Baseline demographic and ocular characteristics were similar between the groups included in the final analysis (Table 1).

**Fig. 1:**
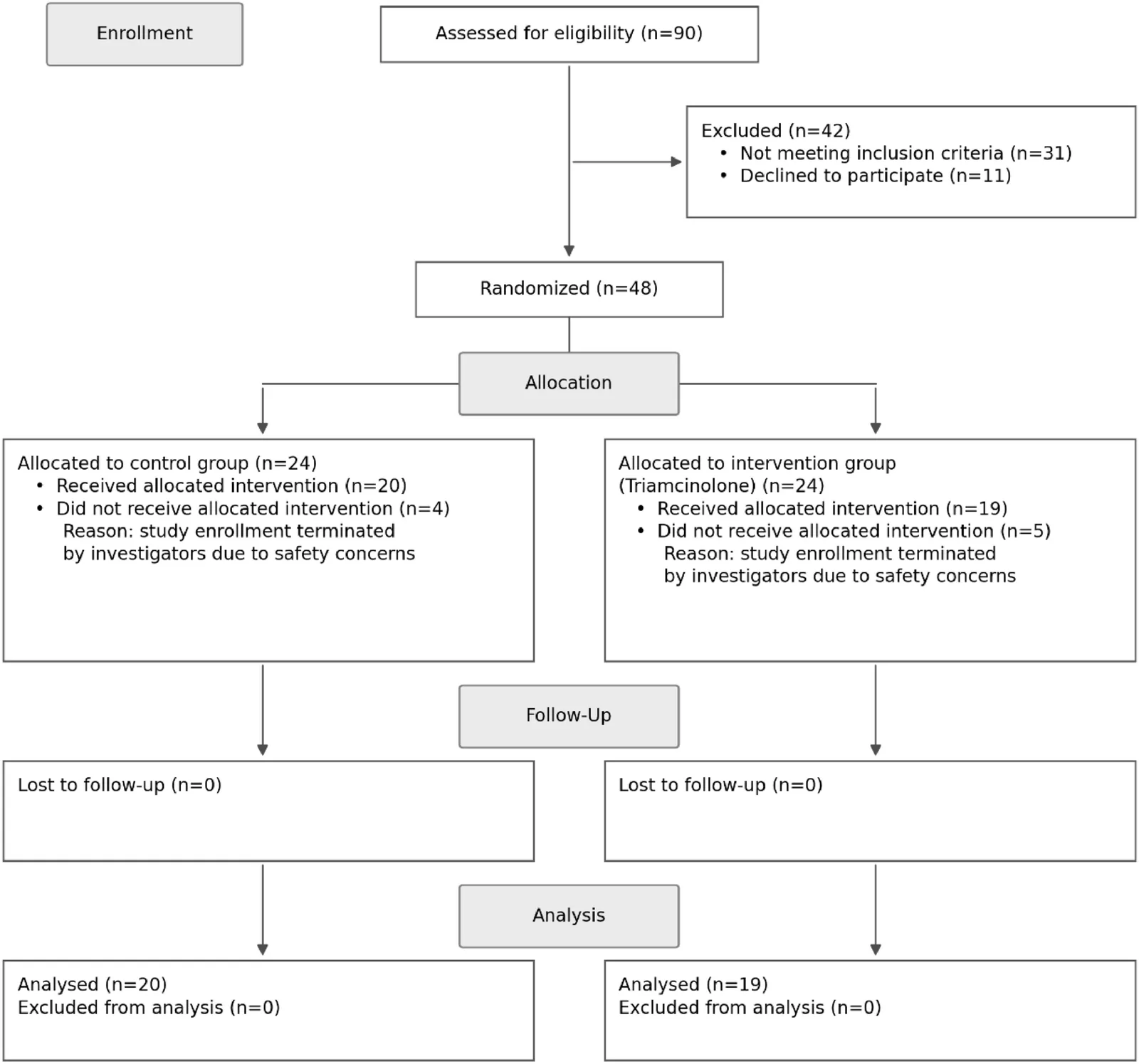
CONSORT 2010 flow diagram detailing participant progress through the enrollment, allocation, follow-up, and analysis phases of the randomized trial.

**Table 1:** Demographic and ocular characteristics of the study population.

Variable	Control (n = 20)	Triamcinolone (n = 19)	p-value
Age (years)	63.8 ± 10.84	63.36 ± 11.64	0.90
Gender (Female/Male)	9/11	11/8	0.85
Eye (Right/Left)	9/11	11/8	0.85
Ethnicity (White/Mixed/Black)	6/7/7	7/9/3	0.66/0.44/0.14
Preoperative IOP (mmHg)	24.45 ± 8.03	24.95 ± 6.80	0.83
Preoperative eye drops	3.60 ± 0.50	3.63 ± 0.59	>0.99

Data are presented as mean ± standard deviation or n. No significant differences were found between the groups at baseline (p > 0.05 for all comparisons).

Mean preoperative IOP was similar between groups: 24.45±8.03 mmHg in the control group and 24.95±6.80 mmHg in the triamcinolone-treated group (p = 0.83) After six months, the mean IOP was reduced to 14.05±3.99 mmHg in the control group and 14.33±4.70 mmHg in the triamcinolone group. There was no statistically significant difference in mean IOP between the groups at any evaluated time point (p > 0.05, Two-Way ANOVA), with a mean IOP reduction of 42.53% in the control group and 42.56% in the triamcinolone group after 180-days (p > 0.99 for between-group comparison at 180-days). Mean IOP was similar between groups at all protocol time points (Table 2). However, mean values masked significant differences in distribution (Fig. 2A‒D). At 60-days, the triamcinolone group exhibited wide IOP variance due to the simultaneous occurrence of hypotony and pressure spikes (Fig. 2B).

**Table 2:** Results of Sidak's multiple comparisons test evaluating differences in intraocular pressure between groups; p-values < 0.05 were considered significant.

Day	Control (Mean ± SD)	Triamcinolone (Mean ± SD)	Adjusted p-value
0 (Preoperative)	24.45 ± 8.03	24.95 ± 6.80	>0.999
1	14.35 ± 5.97	14.00 ± 6.20	>0.999
7	15.89 ± 8.18	15.00 ± 7.73	>0.999
14	17.10 ± 4.60	17.00 ± 7.69	>0.999
21	16.42 ± 4.67	21.21 ± 8.32	0.2887
30	19.10 ± 3.92	15.10 ± 5.06	0.1338
60	15.94 ± 6.89	15.05 ± 10.43	>0.999
90	14.05 ± 4.47	13.50 ± 4.87	>0.999
180	14.05 ± 3.99	14.33 ± 4.70	>0.999

Mean intraocular pressure (IOP) at baseline and postoperative follow-up visits. Data are presented as mean ± standard deviation (mmHg). p-values represent the between-group comparison at each time point, adjusted using Sidak's multiple comparisons test.

**Fig. 2:**
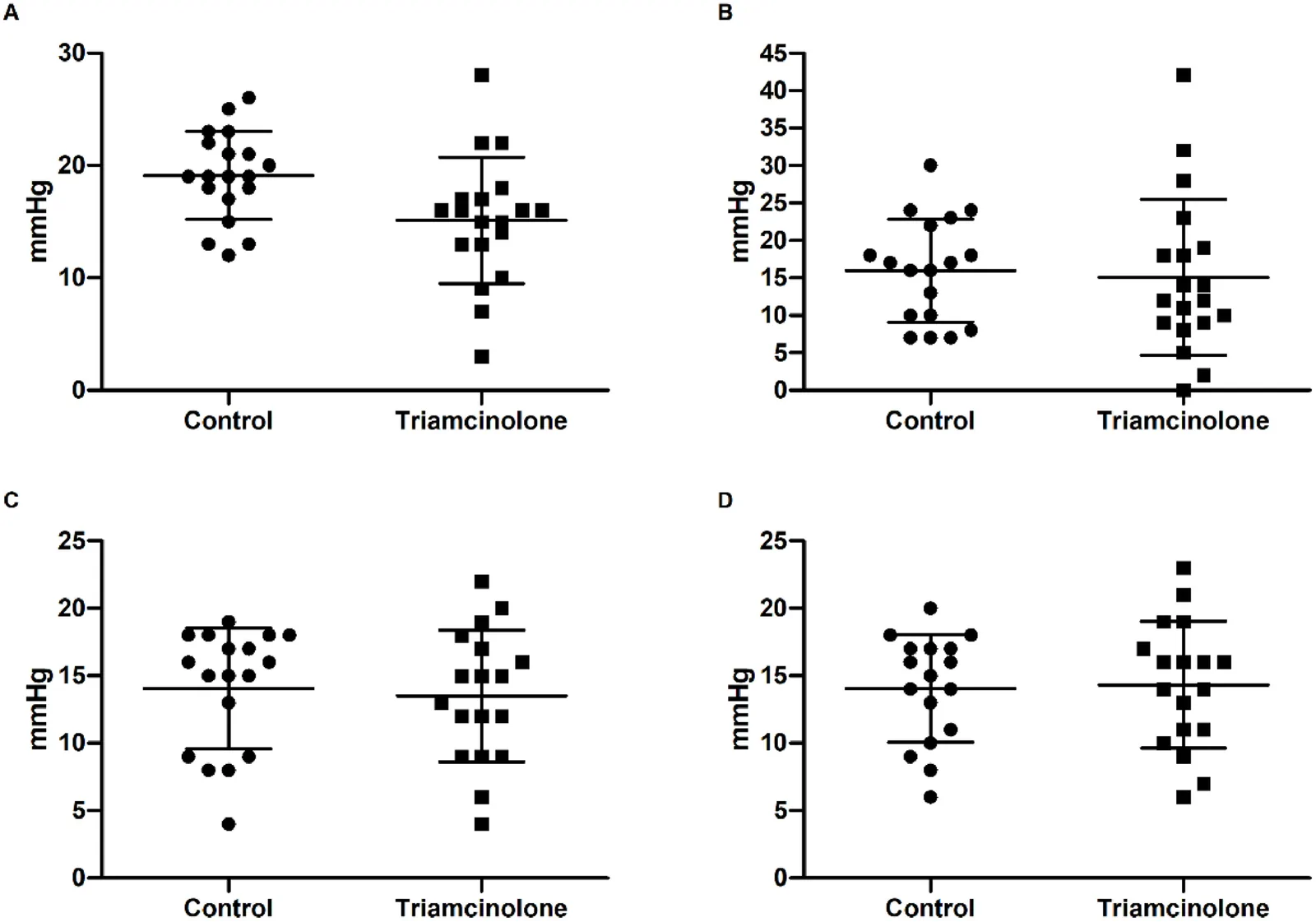
Scatter plots showing the distribution of Intraocular Pressure (IOP) at different postoperative time points. (A) 30-days; (B) 60-days; (C) 90-days; (D) 180-days. Note the wide variance in the Triamcinolone group at 60-days (Panel B), reflecting the occurrence of hypotony in some patients versus pressure spikes in others, compared to the tighter clustering in the Control group.

The mean number of glaucoma medications was also similar preoperatively (control: 3.60±0.50; triamcinolone: 3.63±0.59, p > 0.99). Both groups showed reduced medication use after 6-months (control: 1.57±1.57 medications; triamcinolone: 1.16±1.45 medications), with no statistically significant difference between groups (p = 0.95) (Table 3).

**Table 3:** Number of Eye Drops Used Preoperatively and Postoperatively at 6 Months. Analysis using One Way ANOVA for comparison and Sidak's multiple comparisons post-test, p < 0.05 considered significant.

Day	Control (Mean ± SD)	Triamcinolone (Mean ± SD)	p-value
0	3.60 ± 0.50	3.63 ± 0.59	>0.99
180	1.57 ± 1.57	1.16 ± 1.45	0.95

Qualified success according to Criterion I (5 ≤ IOP ≤ 21 mmHg with or without eye drops) was achieved similarly in both groups (90% in the control group and 89.47% in the triamcinolone group, p > 0.99). Nevertheless, a critical safety signal was observed coinciding with suture dissolution (approx. 6-weeks). In the triamcinolone group, 10 of 19 patients (52.6%) developed late-onset hypotony, compared to 0% in the control group (p < 0.001) (Fig. 3). Of these 10 patients, 7 (36.8%) required surgical re-intervention (anterior chamber reformation or tube ligation) due to clinically significant hypotony. Consequently, the overall rate of serious complications (defined as those requiring re-intervention or threatening vision) was significantly higher in the triamcinolone group (42.1%, n = 8; driven by the surgical hypotony cases and one case of implant extrusion) compared to the control group (5.0%, n = 1; one case of malignant glaucoma resolved medically) (p = 0.008) (Table 4). Other minor complications, such as early corneal edema and transient early hypotony, were similar between groups.

**Fig. 3:**
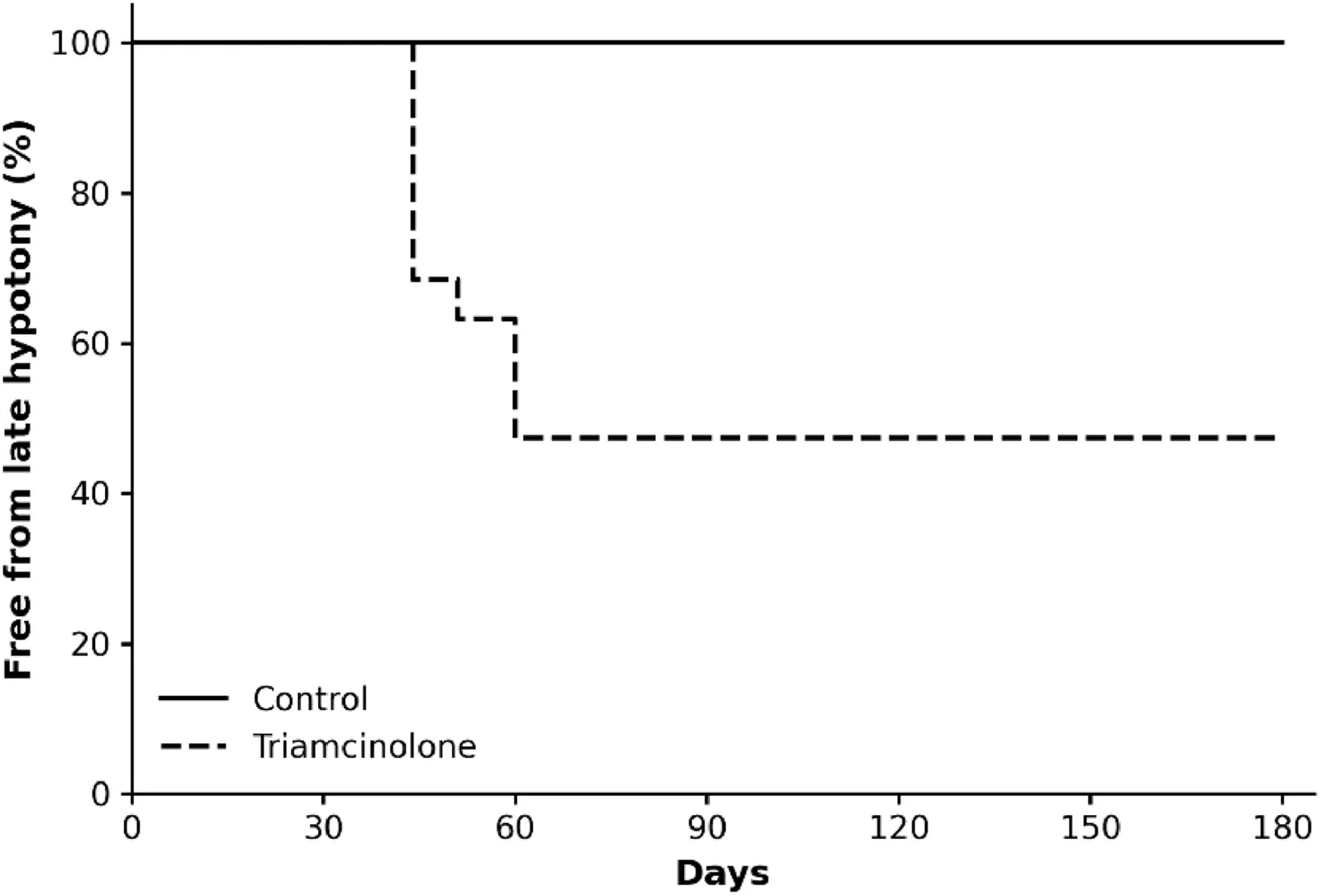
Kaplan-Meier survival curve demonstrating the cumulative probability of remaining free from late-onset hypotony (defined as IOP < 6 mmHg occurring after day 30) over the 6-month follow-up. A significant “step-off” failure rate is observed in the Triamcinolone group starting around day 42 (coinciding with suture dissolution), while the Control group remained stable (Log-rank test, p < 0.001).

**Table 4:** Analysis of Serious Complications Observed Between Groups During the Study.

All Complications	Control (n = 20)	Triamcinolone (n = 19)	p-value
Early Hypotony	2 (10%)	4 (21.1%)	0.41
Early Corneal Edema	3 (15%)	4 (21.1%)	0.69
Anterior Uveitis	1 (5%)	0 (0%)	>0.99
Cataract	0 (0%)	2 (10.5%)	0.23
Late Hypotony	0 (0%)	10 (52.6%)	<0.001
Managed with Observation (not serious)	0 (0%)	3 (15.8%)	0.11
Requiring Surgical Re-intervention	0 (0%)	7 (36.8%)	0.003
Malignant glaucoma	1 (5%)	0 (0%)	>0.99
Implant Extrusion	0 (0%)	1 (5.3%)	0.49
Serious Complication (hypotony requiring re-intervention, malignant glaucoma and implant extrusion)	1 (5%)	8 (42.1%)	0.008

## Discussion

This randomized clinical trial, the first to evaluate adjunctive triamcinolone with a non-valved glaucoma drainage device, demonstrates that while the Susanna implant is effective, the addition of 20 mg of sub-Tenon triamcinolone provides no benefit to IOP control and, critically, leads to a significantly higher rate of sight-threatening hypotony. This finding contrasts with studies on valved devices and suggests a distinct interaction between potent corticosteroids and the uninhibited flow of non-valved implants.

Previous studies on Ahmed and Baerveldt implants have shown considerable IOP reductions. For instance, Ahmed implants have reported preoperative IOPs between 31 and 32 mmHg, with postoperative reductions to approximately 15 mmHg, indicating a 50%‒54% reduction. Similarly, Baerveldt implants have shown preoperative IOPs around 31 to 34 mmHg, with postoperative values ranging from 12 to 14 mmHg, translating to a 55%‒63% reduction. Studies by Gedde et al., Roy et al., and others have documented these reductions extensively.^10^, ^11^, ^12^, ^13^, ^14^, ^15^

Studies on the Susanna implant showed positive results in reducing IOP. Biteli et al. observed a preoperative IOP mean of 31.5 mmHg with a mean reduction to 12.8 mmHg (a mean reduction of 59.36%) after six months of follow-up. In a study conducted by Susanna et al., the preoperative IOP mean was 23 mmHg, with a reduction to 11 mmHg (52.17%) after 12-months of follow-up.^6^^,^^7^

In comparison, our study revealed a mean IOP reduction achieving final mean IOPs of approximately 14 mmHg in both groups (42.53% reduction in controls, 42.56% in the TA group). These percentage reductions are somewhat lower than some reports for Ahmed, Baerveldt, and even other Susanna implant studies, but remain clinically significant. It is important to note that the mean preoperative IOP in our study (around 24‒25 mmHg) was lower than in many cited studies, which may account for the smaller percentage reduction observed. Despite this, the final mean IOP of around 14 mmHg is comparable to many published outcomes for other GDDs, indicating effective pressure management.

Regarding the effect of TA on IOP reduction with the Susanna implant, our findings of similar efficacy between the TA and control groups (approximately 42.5% reduction, final IOP ∼14 mmHg) show that TA provided no additional IOP-lowering benefit. While some studies of TA with Ahmed implants reported substantial IOP reductions (e.g., Yazdani et al. 53.29%; Turalba & Pasquale 42.85%), the key finding from our RCT is the lack of *added* benefit from TA compared to the GDD alone.

Regarding the use of hypotensive eye drops, our study noted a significant reduction in the number of medications required postoperatively in both groups (to approximately 1.6 in controls and 1.2 in the TA group). This reduction mirrors findings from previous studies on Ahmed and Baerveldt implants, which reported postoperative medication use averaging 1.5 to 2 drops per patient.^10^, ^11^, ^12^, ^13^, ^14^, ^15^ However, the difference in medication use between our TA and control groups was not statistically significant, indicating no clear benefit of TA in reducing medication dependency in this study.

Visual acuity outcomes in our study showed no significant differences between the control and triamcinolone groups at any time point, indicating that triamcinolone did not appear to adversely affect visual acuity in this cohort. This finding is consistent with studies by Yazdani et al. and Pasquale and Turalba, which also reported no significant impact on visual acuity with triamcinolone use.^8^^,^^9^

The qualified success rates (Criterion I: IOP ≤ 21 mmHg) observed in our study were high and comparable between groups (control 90%, TA 89.47%), aligning well with rates reported for the Susanna implant used alone (e.g., Biteli et al. 86%, Susanna et al. 86.4%).^6^^,^^7^

However, our study did reveal a notable difference in complication rates between the groups. The triamcinolone group experienced a significantly higher rate of serious complications (42.1%), including late hypotony (52.63% in TA group vs. 0% in controls, p < 0.001). This finding contrasts with previous studies on Ahmed implants, where triamcinolone did not significantly increase complication rates.^8^^,^^9^ The hypotony observed may result from triamcinolone-induced excessive inhibition of the normal fibrotic capsule formation around the implant plate, potentially leading to overfiltration. Further research, including histopathological analyses, would be required to validate this hypothesis. The complication rate in the control group was comparable to those reported for Susanna implants by Biteli et al. (9%) and Susanna et al. (4.5%).^6^, ^7^, ^8^, ^9^

The decision to prematurely terminate enrollment after 39 eyes was made by the Data Safety and Monitoring Board due to the clear safety signal observed in the triamcinolone arm. While this early termination was ethically imperative, it limited the final sample size relative to the initial target of 48 eyes. This sample size reduction may impact the statistical power for detecting smaller differences in secondary outcomes and should be considered when interpreting these findings.

Our study presents some limitations. The six-month follow-up period limits long-term efficacy conclusions, and the early termination due to severe complications introduces potential bias. Regarding the complications, a critical limitation of our study design was the use of a standard absorbable ligature. As noted by experts, using an occluding intraluminal stent suture (e.g., prolene) that can be removed selectively might have allowed for better titration of flow in the presence of TA, potentially avoiding the hypotony observed with the sudden dissolution of the polyglactin suture. The high rate of hypotony in the TA group highlights the potent antifibrotic effect of the drug on the bleb capsule, which requires a more controlled flow strategy. Future research with larger samples and extended follow-ups is essential. Other limitations should be considered. We did not perform histopathological or imaging evaluations (e.g., anterior segment ultrasound or OCT) to objectively characterize the fibrous capsule around the implant. This restricts our understanding of the precise mechanisms by which triamcinolone may increase hypotony rates. Furthermore, our strict inclusion criteria ‒ limited exclusively to refractory primary open-angle glaucoma ‒ restrict the generalizability of our findings to other types of glaucoma commonly treated with drainage implants, such as neovascular or uveitic glaucoma. Lastly, individual factors potentially affecting responses to triamcinolone treatment (such as advanced age, diabetes, or prior topical steroid use) were not systematically evaluated as predictors of outcome and the tapering of postoperative topical corticosteroids was based on clinical judgment rather than a fixed protocol, which, while reflecting real-world practice, could introduce variability.

## Conclusion

In conclusion, while the Susanna drainage implant demonstrates effective IOP control in this cohort of refractory glaucoma patients, the adjunctive use of sub-Tenon triamcinolone (20 mg) was associated with a significantly increased risk of serious complications, especially hypotony, suggesting it is not a safe adjunct at this dose with the Susanna non-valved implant. These findings reinforce the efficacy and safety profile of the Susanna drainage implant when used as a standalone procedure and do not support the routine adjunctive use of triamcinolone as investigated in this setting. This study provides valuable insights for clinical practice and emphasizes the importance of and highlights the need for caution when considering potent anti-inflammatory adjuvants with non-valved GDDs.

## Clinical trial registration

Name of Registry: *Brazilian Registry of Clinical Trials – ReBEC.*

Registration Number: RBR-3mcfsb9.

Date of Registration: *07/31/2025.*

URL of Registry: https://ensaiosclinicos.gov.br/rg/RBR-3mcfsb9.

## Summary

This randomized trial (n = 39 eyes) found that adjunctive sub-Tenon triamcinolone (20 mg) with the Susanna glaucoma drainage implant did not improve 6-month IOP control but significantly increased serious complications, primarily hypotony, compared to implant alone.

## Declaration of generative AI and AI-assisted technologies in the writing process

The authors declare that no generative AI or AI-assisted technologies were used in the writing process of this manuscript.

## Sources of support/funding

This study received no specific external funding. Dr. Takatsu is a recipient of a PhD scholarship from the Coordenação de Aperfeiçoamento de Pessoal de Nível Superior (CAPES), Brazil.

## CRediT authorship contribution statement

**Marcus Vinicius Takatsu:** Conceptualization, Methodology, Investigation, Formal analysis, Writing – original draft, Funding acquisition. **Karin Chiaradia Takatsu:** Investigation. **Kaio Santos Soares:** Investigation. **Kayo Pinheiro Esposito:** Investigation. **Leonardo Eleuterio Ariello:** Formal analysis, Writing – review & editing. **Renato Antunes Schiave Germano:** Conceptualization, Writing – review & editing. **Marcio Henrique Mendes:** Methodology, Writing – review & editing. **Aline Domingos Pinto Ruppert:** Formal analysis, Writing – review & editing. **Marcelo Hatanaka:** Methodology, Formal analysis, Writing – review & editing, Supervision.

## Conflicts of interest

The authors declare no conflicts of interest.

## Data Availability

The datasets generated and analyzed during the current study are available from the corresponding author upon reasonable request.
